# Supplementary material for: Improved Therapeutic Efficacy of CBD with Good Tolerance in the Treatment of Breast Cancer through Nanoencapsulation and in Combination with 20(S)-Protopanaxadiol (PPD)
Source: Pharmaceutics. 2022 Jul 22;14(8):1533. doi: 10.3390/pharmaceutics14081533 (PMC9332327; doi:10.3390/pharmaceutics14081533)
Supplement: Supplementary file 1 [file pharmaceutics-14-01533-s001.zip › pharmaceutics-1727408-supplementary.pdf]

Table S1. Formulation and particle size of various CBD liposomes and CBD-PPD liposomes.

	Formulation	Size(nm)	PDI
CBD liposomes	SPC:chol:CBD=4:1:1	192.6±2.84	0.209±0.005
CBD-PPD liposomes (CP-liposomes)	SPC:PPD:CBD =4:1:1	138.8±1.76	0.245±0.006
PPD liposomes	SPC:PPD=4:1	113.5±2.39	0.323±0.026
maltose modified CBD liposomes (GMC-liposomes)	SPC:chol:CBD:Mal=4:1:1:1	145.0±2.97	0.270±0.010
maltose modified CBD-PPD liposomes (GMCP-liposomes)	SPC:PPD:CBD:Mal =4:1:1:1	179.3±2.29	0.267±0.018

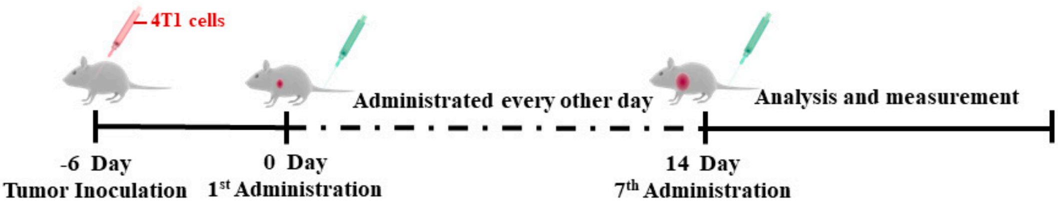

Figure S1. Schematic illustration of treatment of chemotherapy to a 4T1 tumor-bearing mice.

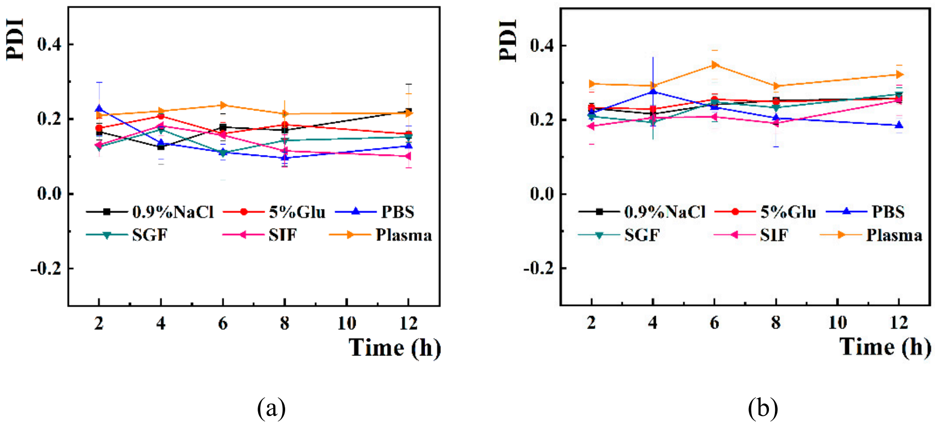

Figure S2: The PDI value of CP-liposome (a) and GMCP-liposome (b) in various physical media at 37°C.

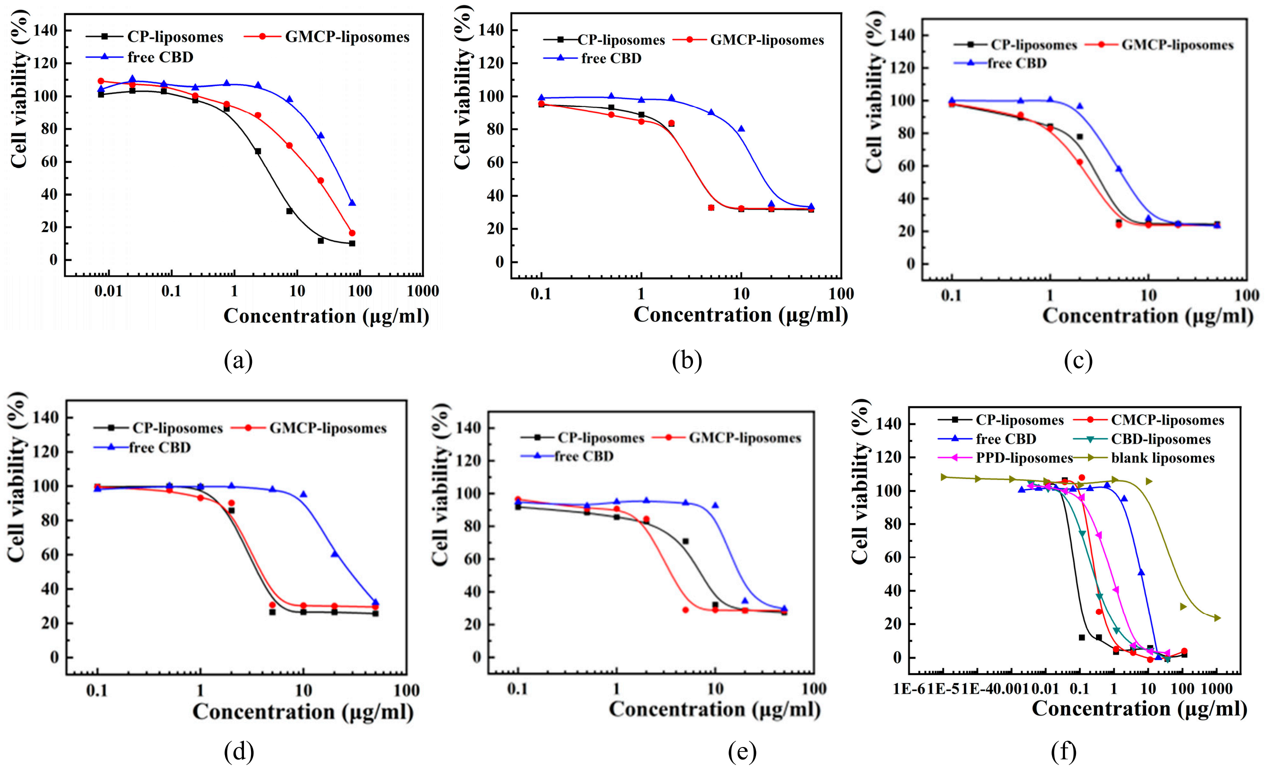

Figure S3: The tumor cell growth inhibition curve of free CBD, CP-liposomes, GMCP-liposomes against MCF-7 cell line (a), A549 cell line (b)，C6 cell line (c)，Hela cell line (d) and HepG2 cell line (e). The tumor cell growth inhibition curve of free CBD, CP-liposomes, GMCP-liposomes, PPD liposomes and CBD-liposomes against 4T1 cell line (f).
